# Neurophysiologically-informed markers of individual variability and pharmacological manipulation of human cortical gamma

**DOI:** 10.1016/j.neuroimage.2017.08.034

**Published:** 2017-11-01

**Authors:** A.D. Shaw, R.J. Moran, S.D. Muthukumaraswamy, J. Brealy, D.E. Linden, K.J. Friston, K.D. Singh

**Affiliations:** a Cardiff University Brain Research Imaging Centre, School of Psychology, Cardiff University, UK; b Department of Engineering Mathematics, Merchant Venturers School of Engineering, University of Bristol, UK; c School of Pharmacy, The University of Auckland, Auckland, New Zealand; d School of Psychology, The University of Auckland, Auckland, New Zealand; e Division of Psychological Medicine and Clinical Neurosciences, School of Medicine, Cardiff University, UK; f Wellcome Trust Centre for Neuroimaging, University College London, UK

**Keywords:** Gamma oscillations, Induced visual responses, Dynamic causal modelling, Intrinsic connectivity, Neuromodulation

## Abstract

The ability to quantify synaptic function at the level of cortical microcircuits from non-invasive data would be enormously useful in the study of neuronal processing in humans and the pathophysiology that attends many neuropsychiatric disorders. Here, we provide proof of principle that one can estimate inter-and intra-laminar interactions among specific neuronal populations using induced gamma responses in the visual cortex of human subjects – using dynamic causal modelling based upon the canonical microcircuit (CMC; a simplistic model of a cortical column). Using variability in induced (spectral) responses over a large cohort of normal subjects, we find that the predominant determinants of gamma responses rest on recurrent and intrinsic connections between superficial pyramidal cells and inhibitory interneurons. Furthermore, variations in beta responses were mediated by inter-subject differences in the intrinsic connections between deep pyramidal cells and inhibitory interneurons. Interestingly, we also show that increasing the self-inhibition of superficial pyramidal cells suppresses the amplitude of gamma activity, while increasing its peak frequency. This systematic and nonlinear relationship was only disclosed by modelling the causes of induced responses. Crucially, we were able to validate this form of neurophysiological phenotyping by showing a selective effect of the GABA re-uptake inhibitor tiagabine on the rate constants of inhibitory interneurons. Remarkably, we were able to recover the pharmacodynamics of this effect over the course of several hours on a per subject basis. These findings speak to the possibility of measuring population specific synaptic function – and its response to pharmacological intervention – to provide subject-specific biomarkers of mesoscopic neuronal processes using non-invasive data. Finally, our results demonstrate that, using the CMC as a proxy, the synaptic mechanisms that underlie the gain control of neuronal message passing within and between different levels of cortical hierarchies may now be amenable to quantitative study using non-invasive (MEG) procedures.

## 1 Introduction

There is increasing evidence that cortical oscillations play a crucial role in distributed neuronal processing across a range of cognitive domains and spatial-scales; from cortical columns to whole-brain networks (Donner and Siegel, 2011). Importantly, oscillations can be specific and sensitive markers of pathophysiology in a variety of clinical conditions (Mathalon and Sohal, 2015) and are sensitive to pharmacological manipulation (Muthukumaraswamy, 2014). Furthermore, evidence suggests that frequency-specific oscillations arise through the interactions of particular neuron types in distinct neuronal circuits, with higher-frequency (>13 Hz *beta* and *gamma*) oscillations generated within laminar-specific regions of cortical macrocolumns, and lower-frequency (1–12 Hz *delta, theta* and *alpha*) oscillations generated over longer ranges, facilitating communication between brain regions (Buzsáki and Wang, 2012).

One simple task-driven cortical oscillation that has received much attention is the sustained visual gamma oscillation. This is typically induced by high-contrast edge stimuli and seen in LFP recordings in primary visual cortex of cat (Gray and Singer, 1989), monkey (Ray and Maunsell, 2010, Gieselmann and Thiele, 2008) and human (Adjamian et al, 2004, Hoogenboom et al., 2006, Muthukumaraswamy and Singh, 2013) magnetoencephalography (MEG). These fast stimulus-bound responses may play a crucial role in functional integration through, for example, facilitating communication through coherence (Fries, 2005). Sustained visual gamma oscillations emerge following an initial stimulus-driven response (at approx. 300 msec following stimulus onset and lasting for as long as the stimulus is present). They are thought to arise within V1 from interactions between superficial pyramidal cells and inhibitory interneurons (the so-called PING model) (Xing et al., 2012, Spaak et al., 2012), where the amplitude and, particularly, frequency reflect the balance between excitation and GABAergic inhibition of coupled neuronal populations. In human recordings, the parameters of the visual gamma oscillation, particularly the narrow-band peak frequency, amplitude and phase stability have been shown to vary across individuals, be robust over multiple testing sessions (Muthukumaraswamy et al., 2010) and be genetically determined (van Pelt et al., 2012).

Taken together, these findings speak to the exciting possibility that gamma oscillations could be used as sensitive biomarkers of synaptic function and, importantly, provide a link between non-invasive human studies and both in-vitro and in-vivo animal models. However, a simple characterisation of the phenomenological features of visual gamma cannot address the underlying synaptic mechanisms. For example, an increase in the amplitude of gamma oscillations following an experimental manipulation could reflect a number of synaptic or network changes. One principled way to move beyond quantifying simple data features (e.g. coherence) is to use mechanistic neurophysiologically-informed models that are fit to observed data to quantify the underlying microcircuitry (e.g., intrinsic connectivity between superficial pyramidal cells and inhibitory interneurons).

Dynamic causal modelling for steady-state responses (DCM-SSR) (Moran et al., 2009) provides a framework for explaining spectral data (i.e. frequency domain oscillations) in terms of network changes within a prescribed generative model. These models are fitted to the observed data (e.g., cross-spectral density) using standard Bayesian procedures to furnish estimates of synaptic parameters and effective connectivity – and the evidence for any particular model. Given a suitable model of visual cortex, DCM affords greater mechanistic insight, relative to conventional physiological signal analyses, potentially enhancing our ability to explain normal variability, characterise pathophysiology and providing higher specificity to detect pharmacological effects. DCM starts with the selection of data features, such as coherence and spectral power, which have proven functionally relevant in several domains (Fries, 2005). These data features are used to inform a physiologically plausible model of neural population responses. Because population responses can be used to generate local field potentials (LFPs) or non-invasive electrophysiological responses (EEG or MEG), DCM provides a principled way to integrate data from both animal and in-vitro experiments, allowing forward and backward translation.

Pioneering work by Douglas & Martin (Rodney et al., 1991) has demonstrated that macro-columns within visual cortex exhibit a repeated pattern of microcircuits with intrinsic laminar-specific architecture, each of which can be modelled in terms of ‘canonical’ interactions among a small number of neuronal populations. This work revealed that the ensuing canonical microcircuits could reproduce frequency-specific responses obtained through local-field recordings; demonstrating that canonical models are both biologically realistic and have the architecture necessary to perform the computations required by visual processing. Subsequent work demonstrated that these microcircuits are relatively preserved across much of the cortex, leading to the development of a canonical microcircuit (CMC) (Douglas et al., 1991). Subsequent studies in animals have further refined and extended the architecture and computational characteristics of the CMC (Haeusler and Maass, 2007, Bastos et al, 2012).

Moran et al. have provided evidence for the biological validity and accuracy of dynamic causal modelling with the canonical microcircuit model through pharmacological manipulation of key model parameters using isoflurane (Moran et al., 2011a, Moran et al., 2011b) and ketamine (Moran et al., 2014) in rodents. Using pharmacological manipulations enabled a validation of DCM in this context, since inferred changes in microcircuit properties could be corroborated by the known physiological effects of pharmacological interventions. More recently, Muthukumaraswamy et al., (2015). demonstrated similar effects using ketamine in humans, while Gilbert et al., (2016). demonstrated the sensitivity of DCM models to single-gene mutation ion channelopathies in human case studies.

Here we use a canonical microcircuit model of V1 macro-columns to explain individual variability in MEG data obtained from a normative sample of 97 subjects, obtained during a visual grating paradigm. Model inversion or fitting demonstrated stability of model parameters across subjects and identified a key role for inhibitory gain control parameters in determining gamma frequency and amplitude. Moreover, we demonstrate that the inhibitory interneuron time constant – also identified as a key determinant of gamma frequency – was sensitive to the GABA re-uptake inhibitor tiagabine.

## 2 Materials and methods

In Experiment 1, the normative cohort study, Participants were 97 healthy control volunteers (mean age = 24.0 years, sd = 4.5 years, 35 male, 62 female) and were scanned as part of a larger study involving several MR scan protocols and MEG experiments. Only the visual gamma experiment and anatomical MRI are presented in this study.

In Experiment 2, the tiagabine study, data were analysed from a previously published (Muthukumaraswamy et al, 2013, Magazzini et al, 2016), pharmacological manipulation study using the GAT-1 reuptake inhibitor, tiagabine, an anti-epileptic drug that is known to raise the synaptic availability of GABA. The full details of the participants and experimental protocol are reported in Muthukumaraswamy et al., (2013). Fifteen healthy volunteers took part in a single-blind, placebo-controlled, crossover study. The study was divided into two days, each comprising four sessions: First a “pre” MEG measurement session, followed by oral administration of either placebo or 15 mg of tiagabine. Three subsequent ‘post’ MEG measurements were then performed at 1, 3, and 5 h post administration.

In all cases, informed consent was obtained and the studies were performed under ethical approval from the School of Psychology Ethics Committee at Cardiff University for Experiment 1 and the UK National Research Ethics Service (South East Wales) for Experiment 2.

The MEG data for both experiments were recorded using a 275-channel CTF axial gradiometer system (VSM MedTech), located inside a magnetically shielded room. An additional 29 reference channels were recorded for noise cancellation purposes and the primary sensors were analysed as synthetic third-order gradiometers (Vrba, 2001). The sampling rate was 1 200 Hz (0–300 Hz bandwidth). Three electromagnetic coils were placed at set fiducial locations (nasion, left and right pre-auricular) and their position relative to the MEG sensors was localised before and after the session. For source-localization purposes, the MEG data were co-registered to the individual anatomical MRI of each participant by marking the MRI voxels corresponding to the position of the three fiducial coils. The individual anatomical MRIs (1-mm isotropic, T1-weighted FSPGR) were acquired using a 3.0 T MRI scanner (General Electric).

In both experiments, MEG data were collected while participants performed a visual paradigm known to induce strong gamma responses in occipital cortex (Muthukumaraswamy and Singh, 2009). The visual stimulus comprised vertical, stationary, maximum contrast, square-wave gratings with a spatial frequency of 3 cycles per degree, covering 8 × 8° of visual angle and presented on a mean luminance background. In Experiment 1, the grating was presented centrally, with a central red fixation dot, for a randomised duration between 1.5 and 2 s and was followed by an interval of 2 s. In Experiment 2 the stimulus was identical in spatial form, but was presented in the lower-left visual field with a red fixation dot in the top right corner and was presented for a randomised duration of between 1 and 1.5 s and an inter-trial interval of 1.5 s.

Participants were instructed to fixate the red fixation dot and to press a button once the grating disappeared. A warning would be presented if no response was detected within 750 ms. The paradigm consisted of 100 trials in Experiment 1 and 120 trials in Experiment 2, for a total duration of ∼10 min. The stimulus presentations were programmed in Matlab (The MathWorks) using the Psychophysics Toolbox (Kleiner et al., 2007). Stimuli were displayed on a Mitsubishi Diamond Pro 2070 monitor operating at a refresh rate of 100 Hz.

For each dataset, the individual trial epochs were visually inspected and trials containing large artefacts (e.g., head movements, muscle clenching and eye blinks) were excluded. In Experiment 1 an average of 10% of epochs were rejected. In Experiment 2, as the design involves a repeated measures pharmacological study, the number of trials included in the analysis of each session was equalised by removing trials from the end of each recording. This resulted in an average number of trials for analysis of 105.5 per participant (range 82–117).

Beamformer source localization was performed using the Synthetic Aperture Magnetometry (SAM) beamformer approach (Robinson and Vrba, 1999). The difference in gamma power (30–80 Hz) between stimulus and baseline was calculated with a paired t-statistic at each voxel location and virtual sensors were generated at the peak voxel location in the occipital lobe, for each participant and each session separately. It is these estimated time courses that are then taken forward for DCM analysis.

### 2.1 Spectral estimation and modelling using DCM-SSR

Neurophysiologically informed modelling was performed using Dynamic Causal Modelling for steady-state responses (DCM-SSR), as instantiated in the SPM8 package (Moran et al., 2009). DCM uses a generative model approach, coupling a simplistic model of the proposed neuronal activities underlying a signal (*f*), with an observation model (*g*) such as a leadfield weighting (equation (1)).

(1) $$\{\begin{array}{c} \;y=g\left(x,\varphi \right)+\;\varepsilon \\ \dot{x}\;=f\left(x,u,\theta \right) \end{array}$$

We chose a variation on the canonical microcircuit (CMC) as a neural model (see Fig. 2), which contains 4 layer-resolved interacting populations of cells. The CMC estimates the membrane potentials (x_v_) and postsynaptic currents (x_i_) of cell populations through (parameterised) time differential equations of the form:

(2) $$\begin{array}{c} {\dot{x}}_{v}=x_{i}\\ {\dot{x}}_{i}=\;TU-2Tx_{i}-\;T^{2}x_{v}\;\\ U=\left(S\cdot d\right)+\;G+E \end{array}$$

The parameters of these equations include population time-constants (T), local (G) and extrinsic (A) synaptic connectivity strengths, exogenous input (C) strength, delay (D) and presynaptic firing (S). Both the intrinsic connection strengths and population time-constants are of particular interest in the current study, given the importance of excitation-inhibition interactions in generating oscillatory activity at specific frequencies.

The anatomy of the CMC sees excitatory pyramidal populations in superficial and deep cortical layers, separated by excitatory stellate cells in granular layer 4. Finally, a single inhibitory interneuron population resides across the layers. While this may be a simplification of the true cytoarchitecture, the model strikes a balance between biological veracity and model estimability. Adding additional populations (as per Haueslaer and Maess 2007), the model parameter space would expand such that it would be hard to get a robust solution. This balance between complexity and estimability was guided by the extant DCM literature using canonical microcircuits (Muthukumaraswamy et al., 2015; Bhatt et al., 2016; Pinotsis et al., 2013; Auksztulewicz and Friston 2015; Giltbert and Moran 2016; Moran et al., 2011a, Moran et al., 2011b).

The local synaptic connectivity between the 4 populations (Fig. 2) includes reciprocal connections between the interneuron population and each of the 3 excitatory populations. Two non-reciprocal connections exist, both of which are excitatory; one from the L4 stellates to L2/3 pyramidal cells and the other from L2/3 pyramidal to L5/6 pyramidal cells. Finally, each population has its own inhibitory, self-modulatory (gain) connection (G1, 4, 7, 10). Due to this complex coupling, predicting the effect of one parameter on another is non-trivial. The reciprocal L2/3 pyramidal-interneuron parameters (G11, G12) correspond, physiologically, to the generators of the gamma rhythm under the PING model (Tiesinga and Sejnowski 2009; Bartos et al., 2007).

DCM-SSR extends the time differential equations of the CMC (which essentially generate a time course of voltages and postsynaptic currents) with the addition of a transfer function to the frequency domain. Briefly, this entails linearising the equations and calculating a transfer function using the Laplace transform (see Friston 2004).

This frequency-domain model output can be compared with the real spectral density obtained from the virtual sensor data, and the parameters of the model optimized to best fit the model spectral output to the data spectra. This fitting is performed using standard Bayesian inference procedures (variational Laplace) within DCM, allowing both the prior specification of precision on the parameters and an assessment of the covariance of the posterior estimates of the parameters. One of the key strengths of this DCM approach is that each fit of the model generates an estimate of the log-evidence of the model, allowing a principled approach to model comparison. Here, we do not perform model comparison because our primary interest was in intersubject and pharmacological variations in model parameters under the canonical microcircuit model of a single source.

**Fig. 1:**
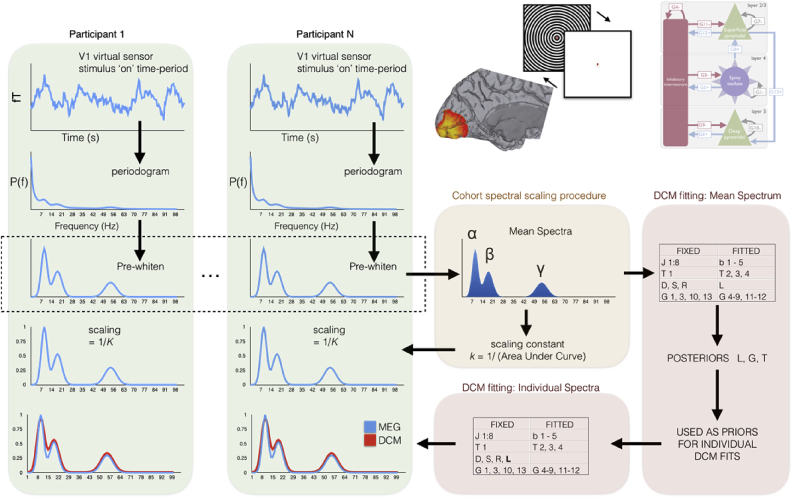
Flowchart demonstrating the analysis pathway for both Experiments. SAM beamforming is used to identify the peak location of gamma responses for each participant and each session (a). This allows a virtual sensor recording to be estimated at this location, and spectral density estimates constructed for the stimulation time (green columns). These spectral density functions are pre-whitened and then normalised so that the global mean spectra have an area-under-the-curve of 1, but individual relative differences in amplitude are preserved. Model spectra are then generated by the canonical microcircuit model (CMC) shown in (b) and the DCM fitting procedure generates model spectra (shown in red) that best fit the true spectral density for each participant/session, shown in blue.

Fig. 1 shows a schematic of the analysis approach we have used for both experiments, which is a modified version of the standard approach in SPM8. Each V1 virtual sensor estimate has its spectral density estimated using a standard Fourier approach using the smoothed periodogram. In order to optimize the fitting algorithm, we then transform this spectral density to remove the strong power-law that dominates brain signals. This ‘pre-whitening’ procedure was done using the approach in Manning et al., 2009; in which a straight-line is fitted, using robust fitting, to the spectral density after transform to log-log space. This power-law is removed from the spectral density to pre-whiten or ‘flatten’ the spectrum, disclosing the presence of alpha, beta and gamma peaks. Interestingly, in invasive LFP recordings, Manning et al., 2009 found that the parameters of this noise function (intercept and gradient) appear to contain neurophysiologically relevant information and were correlated with spiking rates of neurons in the same region (Spaak et al., 2012). We have found that this pre-whitening procedure allows the DCM-SSR approach to robustly fit the spectral density across the 1–100 Hz range we are interested in, presumably because they are more clearly separated from the underlying noise function. In the default version of the DCM-SSR procedure in SPM8, this noise power-law function is estimated at the same time as the model parameters and we retain this model component during fitting, albeit with a prior-specification of a flat ‘white’ noise floor. In one sense, therefore, our pre-whitening approach can be seen simply as helping the inference stage of the DCM-SSR by making sure the true values of non-specific (noisy) fluctuations are approximate prior assumptions.

**Fig. 2:**
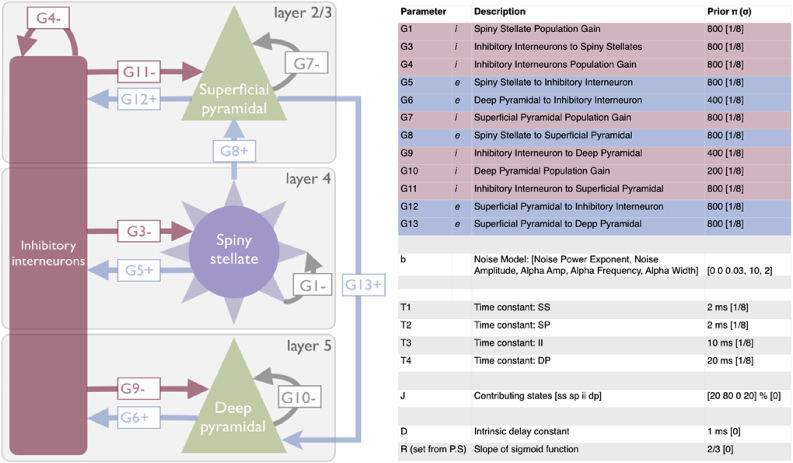
Description of the canonical microcircuit (CMC) used in the DCM procedure. The diagram on the left shows the three-layer model, with excitatory connections shown in blue and the inhibitory (i.e. GABAergic) connections shown in red. Grey arrows represent self-inhibition within each of the excitatory cell populations. On the right are described all of the parameters that define the model, including their prior values (PI) and their precisions (sigma).

Our approach to amplitude scaling of the spectral density also differs from the default scheme in SPM8 (Moran et al., 2009). Amplitude scaling is necessary as real neurophysiological data can be generated with any arbitrary scale, depending on the measurement technique used, and we need to ensure that the CMC model output is in the same range. In our procedure, we wish to preserve the relative differences in spectral amplitude between participants and/or conditions, so we scale each individual spectral density such that the mean spectral density has a sum (or ‘area under the curve’) of 1. This insures that the spectral densities are in a reasonable range that can be matched to the CMC model output, but across-participant and across-condition differences are preserved and, hence, have to be explained by the parameters of the model.

We derive initial starting values for each of the model parameters by first fitting the CMC model to the mean spectral density across participants/conditions. These priors are used in the individual DCM-SSR fits for each of the individual datasets. An initial analysis of the stability of the parameters, performed by fitting the DCM to the model spectra revealed that several of the model parameters had little or no effect on the fitted spectral density. These were G1, G3, G10 and G13. In order to reduce model complexity, these parameters were therefore fixed to their prior means for subsequent analyses. In addition, we found that small changes in T1, the time-constant for the layer-4 excitatory interneurons; i.e., the spiny stellates, had a profound impact on model stability, with small changes in the time-constant inducing phase transitions in spectral output. This parameter was fixed in subsequent analyses.

**Fig. 3:**
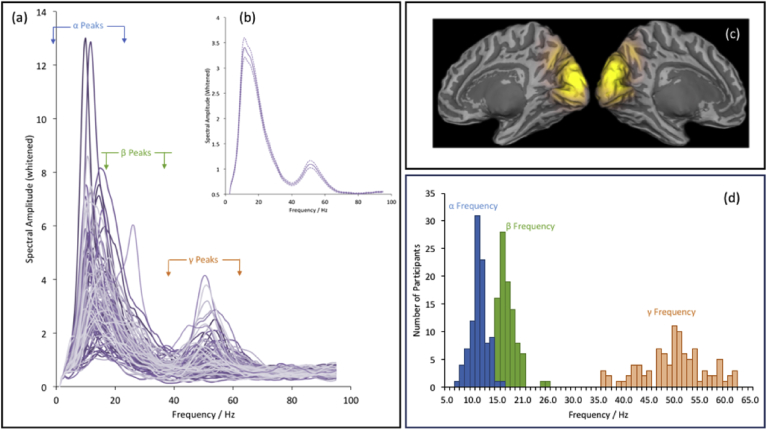
The data features extracted from primary visual cortex beamformer (panel c) virtual electrodes in Experiment 1, the cohort study. Plot (a) shows the whitened spectral amplitude for each of the 97 participants. The response is localised to posterior medial visual cortex (c). For spectra extracted from these peak locations, clear alpha, beta and gamma peaks are present for most participants and are more clearly revealed after the pre-whitening procedure. In the inset (b) the average spectra across all participants is plotted, with dotted lines indicating the standard error on the mean. Note how individual variability in the peak frequency of alpha and beta leads to a merging of these responses in the mean-average spectrum. Finally, in (d) the distribution of peak alpha, beta and gamma frequencies are plotted as separate histograms. Note that the alpha peak is calculated from the DCM fitting procedure in which alpha is explicitly modelled as a Gaussian (see methods), whereas the beta and gamma peaks are extracted from the search for the peak spectral amplitude in their respective ranges.

Finally, we explicitly model the presence of an alpha frequency peak within each spectral response. For the visual experiments analysed here, inspections of the spectral density reveal clear alpha, beta and gamma peaks during the stimulation period, superimposed on a strong noise power-law that we model explicitly (see Fig. 3). The CMC model can generate clear gamma and beta response peaks, consistent with animal evidence that shows that gamma arises in the superficial layers of V1 (2/3), while beta occurs in the deeper layers (5/6) (Roopun, 2010, Haenschel et al., 2000, Buffalo et al., 2011, Kramer et al, 2008, Roopun, 2008). However, a single source model cannot simultaneously generate an alpha peak. This is consistent with evidence that alpha is generated over more extended (multiple source) neuronal networks, including thalamo-cortical ‘loops’ (Vijayan and Kopell, 2012, Bastos et al., 2014). In further work, it may be possible to build extended networks to fully explain the data, but here we take a more pragmatic approach – and add a Gaussian function to the SPM8 noise function to model the alpha peak. The mean of this Gaussian is constrained to be between 8 and 13 Hz. The use of our pre-whitening procedure, coupled with this explicit alpha peak modelling allows us to generate clear separated estimates of the alpha, beta and gamma peaks within our data and hence explore which parameters of the CMC determine the parameters of the beta and gamma peaks for each participant and condition.

## 3 Results

### 3.1 The synaptic determinants of induced responses

For each of the 97 datasets, the peak visual response to a passive grating-patch stimulus was localised using a SAM beamformer (Robinson and Vrba, 1999). The spectral density of a virtual sensor time series, calculated at the best-performing voxel in visual cortex, was estimated for the stimulation period (i.e. 0.3–1.5 s). The temporal evolution of responses in this paradigm is well-characterised (Hoogenboom et al., 2006), comprising theta and gamma frequency phase-locked responses (<300 ms) followed by an induced gamma frequency response (300–1500 ms), with only the latter response analysed here.

Fig. 3a demonstrates all spectra after pre-whiting of the data to remove 1/f noise, demonstrating that most participants show clear alpha, beta and gamma peaks at appropriate frequencies. These spectral responses are also evident in the average spectra across participants (Fig. 3b), although the separation of alpha and beta is obscured by individual variability in the peak frequencies of these responses. The distributions of peak frequencies are shown in the histograms in Fig. 3d.

**Fig. 4:**
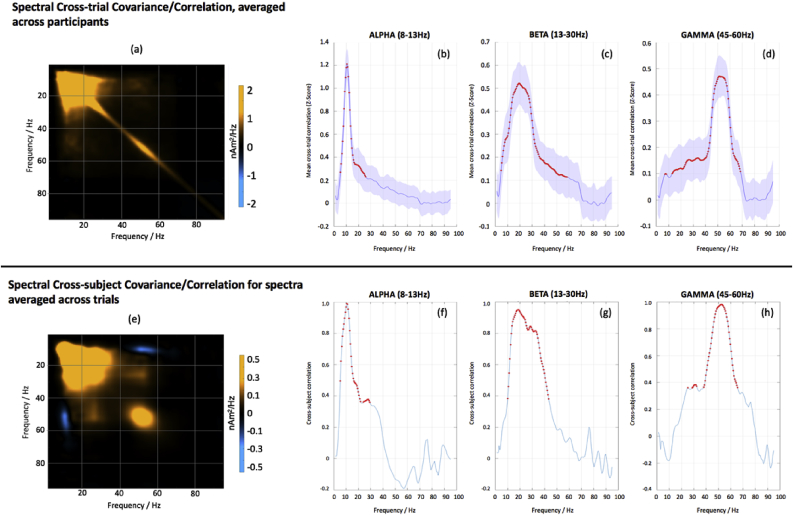
(a) and (e) shows the covariance of the spectral amplitudes, during visual stimulation, calculated either by averaging the mean inter-trial covariance across participants (a) or looking at how the spectral estimates, averaged over trials, co-vary across participants, (e). In both cases the amplitude of the lower frequencies (0–30 Hz) appears correlated across frequencies, whereas the induced gamma response at 40–60 Hz appears less correlated, suggesting separate generative mechanisms. For each of these two analyses, panels b–d and f–h show the cross-correlation of the spectra for alpha, beta and gamma, either across trials (top row) or subjects (bottom row). Red dots demonstrate significant correlations (p < 0.05 corrected for multiple comparisons). Note how correlations are strongest within frequency bands. Panels f–h demonstrate the across-trials correlation after z-transforming and averaging over subjects for the same frequency bands. The shaded bars represent the standard deviation of the Z-score across subjects.

Fig. 4 a–d shows the across-trial spectral covariance map and the associated correlation graphs (after z-transforming and averaging over subjects) for alpha, beta and gamma amplitudes versus all frequencies. Similarly, Fig. 4 e–h demonstrates the across-subject covariance map and associated correlation graphs for alpha, beta and gamma amplitude for spectra that are averaged across trials. In both these evaluations of spectral correlation, the strongest correlations are within-band with limited overlap with other frequency bands. In both cases, although there is some evident overlap of significant correlations at the boundaries between alpha/beta and beta/gamma, this appears of modest magnitude and, in the case of cross-subject correlation, does not extend to a significant correlation between alpha and gamma.

**Fig. 5:**
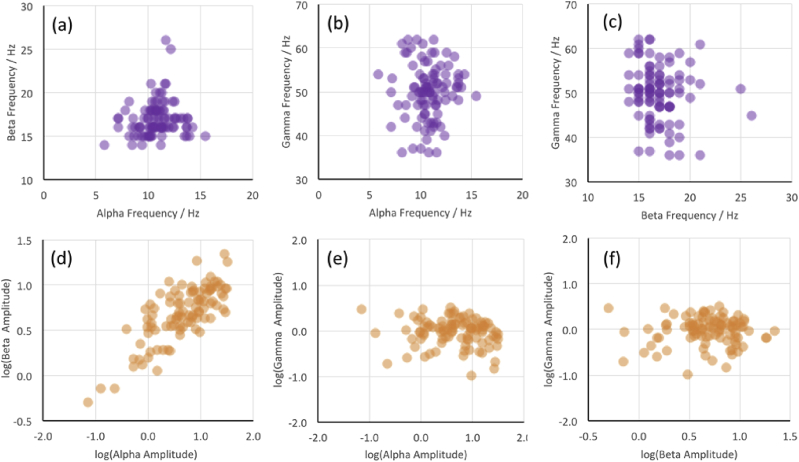
Scatter plots examining the relationships, if any, between peak frequency (top row, a–c) and peak amplitude (lower row, d–f) for the alpha, beta and gamma peaks in the stimulus response. The only apparent relationship is between the alpha and beta amplitudes, consistent with the covariance spectra shown in Fig. 3c/d.

The relative lack of correlation/covariance in Fig. 4 suggests a possible distinct and independent generating mechanism for sustained gamma, compared to alpha/beta, in the visual cortex of our participants. This is shown explicitly in the scatter plots in Fig. 5, in which it is clear that alpha and beta peak amplitudes/frequencies do not correlate with those of gamma across participants: The only significant correlation found was between alpha and beta peak amplitudes [r = 0.77, N = 97 p < 10^−6^] (Fig. 5d).

In order to characterise the neurophysiological basis of individual variability generating these data features, the whitened spectral estimates for each of the 97 participants was analysed using the DCM procedure outlined in Fig. 1. In this analysis, the spectral profile of endogenous neuronal fluctuations and alpha peak were modelled explicitly using mono-exponential and Gaussian functions, respectively. In contrast, the beta and gamma peaks were generated by the synaptic rate constants and intrinsic connectivity of the cortical microcircuit shown in Fig. 2.

### 3.2 Model dependencies

After fitting the DCM to grand averaged data, we identified the most likely value of the model parameters (i.e., synaptic rate constants and intrinsic connectivity). Using these posterior expectations, we then examined the effects of small variations in each parameter on steady-state responses. This is known as a sensitivity or contribution analysis.

This analysis revealed the effects that each model parameter had on the spectral features of the model output (beta and gamma frequency and amplitude, see Table 2). Notably, only parameter G7 (SP→SP) increased gamma frequency while G6 (DP→II) and T4 (DP) increased beta frequency. Parameters increasing gamma amplitude included G4 (II→II), G5 (SS→II), G8 (SS→SP), G11 (II→SP), G12 (SP→II) and T3 (II) while T2 (SP) decreased gamma amplitude. Parameters increasing beta amplitude included G5, G6, G8, G9 (II→DP), G12 and T2, while parameters decreasing beta amplitude included G4, G7, G11 and T3.

**Table 1:** Pearson correlation coefficients between the gamma/beta data features and model parameter estimates. All correlations shown are at least p < 0.05, Bonferroni corrected for multiple tests. Most significant correlations are indicated by a number of asterisks i.e. *: p < 10–3, **: p < 10–4, ***: p < 10–5.

Parameter	G4: ii > ii	G7 sp > sp	G11 ii**→**sp	G12 sp**→**ii	G8 ss**→**sp	G5 ss**→**ii	G6 dp**→**ii	G9: ii**→**dp	T2 sp time	T3 ii time	T4 dp time
Beta Frequency							0.59***				−0.54***
Beta Amplitude	0.55***		−0.38	0.68***	0.36	0.63***	−0.44*	0.82***	−0.35	0.36	
Gamma Frequency		0.64***							0.44*	−0.55***	
Gamma Amplitude			0.80***		0.47**	0.36	0.37			−0.38

**Table 2:** Coupling parameter and time-constant parameter contribution analysis. Arrows represent increase or decrease in given spectral property with increase in parameter value (by 0.1 on log scaling parameter).

Parameter	Description	Beta Frequency	Beta Amplitude	Gamma Frequency	Gamma Amplitude
*Effect of increasing coupling parameter on output spectra*					
G4	II → II (I/gain)		**↓**		**↑**
G5	SS → II (E)		**↑**		**↑**
G6	DP → II (E)	**↑**	**↑**		
G7	SP → SP (I/gain)		**↓**	**↑**	
G8	SS → SP (E)		**↑**		**↑**
G9	II → DP (I)		**↑**		
G11	II → SP (I)		**↓**		**↑**
G12	SP → II (E)		**↑**		**↑**
*Effect of increasing time-constant on output spectra*					
T1	SS	Model unstable to even moderate changes in parameter value			
T2	SP		**↑**		**↓**
T3	II		**↓**		**↑**
T4	DP	**↑**			

Parameters affecting both beta and gamma amplitude in the same direction included G5 (increase), G8 (increase) and G12 (increase), while parameters demonstrating opposite effects in these bands included G4, G11, T2 and T3.

### 3.3 Key determinants of beta and gamma oscillations in the visual cortex

**Fig. 6:**
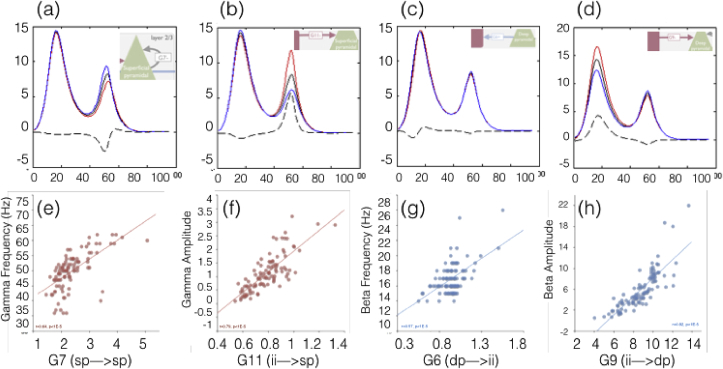
An illustration of how the CMC model parameters explain gamma and beta stimulus responses in primary visual cortex for Experiment 1. Four connection strength parameters were the key determinants of beta/gamma features and are presented here. In the top row (a–d) The black line shows the mean CMC output and demonstrates that the model can generate clear beta and gamma peaks in its spectral output. A contribution analysis shows how small changes in each parameter affect the spectral output of the CMC (here the mean across participants). The red curves show how a small additive perturbation (+0.1) affects this spectral output and the blue lines show the spectral modulation to a small reduction (−0.1). In (a) it can be seen that gamma peak frequency is shifted upwards and downwards by increasing and decreasing the strength of the inhibitory gain control parameter on the superficial pyramidal cells. Note how peak gamma amplitude is also modified by this parameter. In (b) the gamma peak amplitude can be seen to be dependent on the inhibitory drive from the inhibitory interneurons onto the superficial pyramidal cells. Beta frequency is determined by the connection from deep pyramidals onto the inhibitory interneurons (c), whereas beta amplitude is strongly related to the inhibitory drive from the inhibitory interneurons on to the deep pyramidals. In the bottom row (e–h), the relationship between these same model parameters and peak gamma frequency (e), peak gamma amplitude (f), peak beta frequency (g) and peak beta amplitude (h) are shown. Each dot represents one of the 97 participants. Significant correlations are demonstrated (see text in each plot) that are consistent with the contribution analysis shown in the top row.

The four parameters with the greatest influence on beta and gamma peak amplitude and frequencies are shown in Fig. 6. Consistent with invasive animal neurophysiology, gamma frequency and amplitude were affected most by connections in the superficial layers of the visual cortex: The self-inhibition parameter (G7) that controls the gain of superficial pyramidal cells was the predominant determinant of variability in peak visual gamma frequency (Fig. 6a), with higher values of self-inhibition leading to a higher gamma frequency. The key parameter determining gamma amplitude (Fig. 6b) was the strength of the inhibitory connection (G11) between the inhibitory interneuron population and the superficial pyramidal cell population, with stronger inhibition leading to higher gamma amplitude. In contrast, beta amplitude and frequency were linked to connections with the deep pyramidal cell population, again consistent with animal LFP recordings: beta frequency was related to the excitatory drive from the deep pyramidal cells on to the inhibitory interneurons (Fig. 6c, G6), while peak beta amplitude was positively correlated with the strength of the inhibitory connection from the inhibitory interneuron population to deep pyramidals (Fig. 6d, G9). This pattern of influences reflects a similar role of same inhibitory connection to the superficial pyramidals (G11) that are a predominant determinant of gamma amplitude.

In addition to the parameters described above, gamma amplitude was also correlated with the strength of the excitatory drive from the layer 4 spiny stellate cells to both superficial pyramidal cells (G8) and inhibitory interneurons (G5). This may reflect the fact that stimulus input from the LGN mostly arrives in layer 4 stellate cells and so these connections reflect coupling of stimulus drive to oscillatory responses.

The key CMC model parameters include excitatory (+), inhibitory (−), and gain/self-modulatory intrinsic connectivity between populations as well as synaptic rate or time-constants (TC) for each population. Bonferroni corrected correlational analyses of the posterior CMC model parameters (Fig. 6) and beta and gamma spectral features revealed significant predictors (Table 1). The significant correlations are not surprising, because these data features inform posterior estimates. However, their specificity illustrates the distinct synaptic mechanisms that are responsible for the genesis of different frequency responses.

Peak gamma amplitude was predicted by glutamatergic afferents from layer 4 spiny stellate (SS) populations to both superficial pyramidal (SP) and inhibitory interneuron (II) populations as well as by GABAergic afferents from II to SP populations. Gamma frequency was predicted by the TC of SP and II populations as well as by the GABAergic self-modulation of SP populations. Beta amplitude was predicted by all intrinsic connectivity and time constants with the exception of SP self-modulation and deep-pyramidal (DP) population time-constants. Beta frequency was predicted by the glutamatergic connection from DP to II populations and the time-constant of DP populations.

The absolute value of the intrinsic connections (G) can be interpreted in terms of rate constants (i.e., the units are in hertz). In other words, effective connectivity in dynamic causal modelling quantifies the influence of one neuronal state on the rate of change of another. Note that the free parameters used in DCM are log scale parameters that enable the connection strength to be scaled up or down from its prior expectation (these are the values reported in the figures). Similarly, synaptic decay time constants represent temporal dynamics of each population (i.e., the units are in milliseconds). The priors for the superficial pyramidal cells, spiny stellate, inhibitory interneurons and deep pyramidals had prior values of 2, 2, 10 and 20 ms respectively. After DCM fitting, the posterior mean across the population of 97 healthy controls was 0.8, 1.5, 3.1 and 14.3 ms respectively. In general, therefore, the resultant dynamics were faster than their prior expectations based on the literature and previous DCM studies. The biggest difference was in the time-constant of the inhibitory interneurons. However, a study of fast-spiking parvalbumin interneurons in slices taken from adult rat neocortex found that these had time constants that were more rapid than juvenile animals, with specific time constants of 2.6 and 5.9 ms for postsynaptic currents and potentials respectively (Galarreta and Hestrin, 2002). These are close to the mean of 3.1 ms we obtained for the DCM modelling of this human cohort.

### 3.4 Pharmacological intervention

In Experiment 2, 15 healthy volunteers completed a similar MEG visual stimulation protocol before and at 1, 3 and 5 h post oral administration of placebo and the GABA transaminase inhibitor tiagabine (totalling 8 scans each subject). The same DCM-SSR modelling procedure, using the same CMC as the cohort study described above, was applied separately to all (4 × 2) sessions for each of the 15 participants. Drug effects were analysed using a repeated measures ANOVA (implemented in JASP, https://jasp-stats.org) with two within-subject factors, namely *Drug* (two levels: Placebo and Tiagabine) and *Time* (four levels: Pre, 1hr, 3hr and 5hr). A drug effect in this analysis is therefore represented by a significant *Drug x Time* interaction. All the data features relating to alpha, beta, and gamma were subject to this analysis, as were all of the DCM-SSR intrinsic connection strengths and synaptic time constants.

**Fig. 7:**
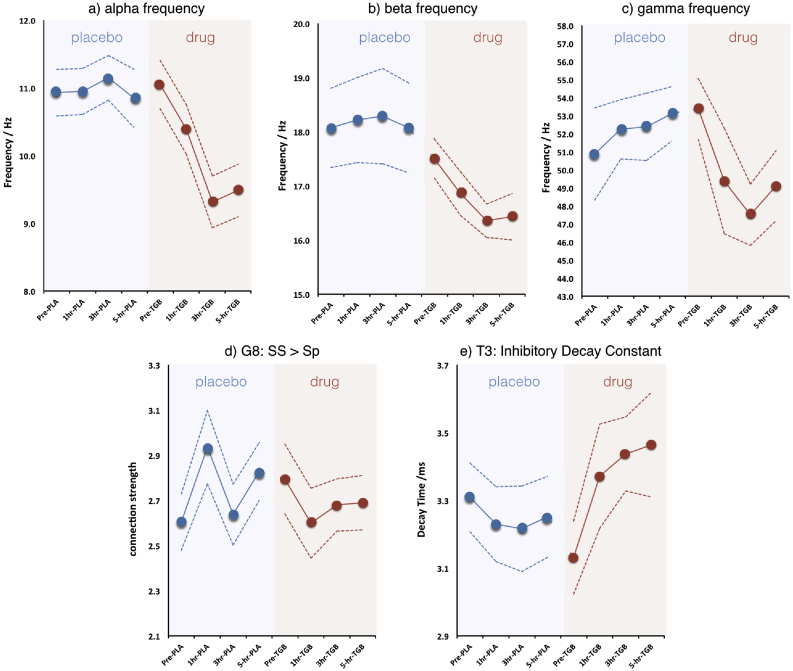
Graphs showing the session/time evolution of the 5 parameters, averaged across all participants, showing a significant Drug × Time interaction in the ANOVA shown in Table 2. Blue dots/symbols show the temporal evolution within the Placebo session, whilst red dots/symbols show the same four time points for the tiagabine (i.e. drug) session. Dotted lines shown ± one standard error on the mean across participants. In (a–c) it is apparent that all three frequencies (alpha, beta, gamma) show a pronounced slowing of the peak frequency, with the biggest effect occurring 3 h post tiagabine administration.

Seven parameters demonstrated an apparent *Drug x Time* interaction when assessed at the p < 0.05 level (uncorrected for multiple comparisons). These were the three parameters specifying the Gaussian model of the alpha peak (peak frequency, peak amplitude and Gaussian width), peak beta frequency and peak gamma frequency (Fig. 7). In terms of the model parameters, the excitatory connection (G8) from the spiny stellates to the superficial pyramidals showed a drug-related reduction and the time-constant of the inhibitory interneurons showed an increase. However, once a correction for non-sphericity (Greenhouse-Geisser) was applied only five parameters demonstrated a significant *Drug x Time* interaction effect. These are reported in Table 3.

**Table 3:** Results of the repeated measures ANOVA demonstrating the three data features and two model parameters that showed a significant Drug × Time interaction after Greenhouse-Geisser correction. Alpha, Beta and Gamma frequencies all showed an effect, as did two of the CMC model parameters. Note that, for some parameters, there are a reduced number of participants because the parameter could not always be successfully identified in all 8 sessions for all 15 participants (a prerequisite for repeated measures ANOVA).

Parameter	Number of participants	F	df (Greenhouse-Geisser)	p
Peak Alpha Frequency/Hz	15	14.35	2.396	<0.001
Peak Beta Frequency/Hz	14	2.940	2.806	0.045
Peak Gamma Frequency/Hz	8	5.74	1.992	0.015
G8, excitatory connection from layer 4 stellates to layer 2 superficial pyramidals	15	3.319	2.291	0.043
T3, inhibitory interneuron decay constant/ms	15	4.671	2.28	0.013

The results of the modelling analysis showed that tiagabine had a pronounced effect in slowing alpha, beta and gamma oscillations. In terms of model parameters, the excitatory drive from the stellates in layer 4 (i.e. input) layer to the superficial pyramidal cells (G8) showed a significant interaction, as did the inhibitory interneuron time constant (T3), which demonstrated an increase in decay time during the tiagabine session. These results are consistent with the cohort modelling in Experiment 1, in which G8 was positively correlated with beta frequency and T3 was negatively correlated with gamma frequency.

Table 4 shows the magnitude of drug-induced changes for those parameters showing a significant *Drug x Time* interaction. In most cases, the biggest effects were seen 3-h after administration of tiagabine.

**Table 4:** Quantification of the magnitude of drug effects. Mean and standard error on the mean (SE) are shown for each session. At 1 h, 3 h and 5 h, the values quoted are changes from the pre-tiagabine session. The significance of these pairwise changes is indicated by * p < 0.05 and **p < 0.0001.

Parameter	Mean (SE) before tiagabine administration	Mean (SE) change at 1-hr post tiagabine	Mean (SE) change at 3-hr post tiagabine	Mean (SE) change at 5-hr post tiagabine
Peak Alpha Frequency/Hz	11.0 (0.4)	−0.7 (0.3)*	−1.7 (0.3)**	−1.6 (0.3)**
Peak Beta Frequency/Hz	17.3 (0.4)	−0.6 (0.3)	−1.0 (0.4)*	−1.0 (0.4)*
Peak Gamma Frequency/Hz	53.4 (1.7)	−4.0 (1.5)*	−5.9 (0.8)**	−4.3 (1.2)*
G8, excitatory connection from layer 4 stellates to layer 2 superficial pyramidals	2.8 (0.2)	−0.2 (0.1)*	−0.1 (0.1)	−0.1 (0.2)
T3, inhibitory interneuron decay constant/ms	3.1 (0.1)	0.2 (0.1)*	0.3 (0.1)*	0.3 (0.1)*

## 4 Discussion

Using neurophysiologically informed modelling we have identified key synaptic parameters accounting for inter-subject variability in peak beta and gamma. These findings replicate preclinical findings, spatially, in terms of their laminar resolved generators, and in terms of the intrinsic connectivity assumed to underlie frequency specific oscillations. Furthermore, we have validated this model against a pharmacological agent with known mechanism of action – demonstrating specificity and sensitivity of the model to subtle perturbations. Crucially, the roles of specific intrinsic connections and population-specific synaptic time constants in generating frequency-specific induced responses was established in the context of empirically optimized values using dynamic causal modelling.

The CMC presented here was informed by electrophysiological studies characterising local circuitry (Rodney et al., 1991, Haeusler and Maass, 2007, Jansen and Rit, 1995). The key difference between our CMC and the default SPM CMC is the addition of reciprocal pyramidal–interneuron connectivity in superficial layers (Thomson et al., 2002). This connection was included in the original model by Douglas and Martin (Rodney et al., 1991, Bastos et al, 2012) and in a recent model of primary motor cortex for modelling movement-related beta oscillations (Bhatt et al., 2016).

The DCM (for MEG) approach has recently received construct validation using genetic (Gilbert et al., 2016) and electrocorticographic models (Phillips et al., 2016). Here, we additionally provide a pharmacological validation. Our approach differed from Phillips et al., (2016), who tested a series of model spaces using Bayesian model selection, and from Gilbert et al. (2016), who used conductance based models for greater (ion channel) biological detail. These differences illustrate that DCMs are valid when using models relevant to the hypothesis at hand. This work differs from other, similar, implementations of the CMC for visual responses (e.g (Gilbert and Moran, 2016).) because we aim to explore the relationship between a parameter set from a fixed (informed) model space and features of the oscillatory response, rather than comparing multi-node architectures underlying ERPs (Gilbert and Moran, 2016) or cross-spectral densities (Moran et al., 2009).

Our finding of laminar separation of beta and gamma generating parameters is consistent with theoretical (Bastos et al., 2012) and invasive animal recordings (Xing et al., 2012, Maier et al., 2010), which report gamma oscillations predominantly arising in superficial layers of cortex through pyramidal – interneuron loops while beta oscillations arise via similar mechanisms but in deep, sub-granular layers. This highlights two important conclusions that follow from our results; namely, that the DCM-CMC framework for MEG data adequately recapitulates laminar-resolved population activity and, more importantly, that induced MEG responses may reflect the output of specific cortical layers. This laminar specificity is important from many perspectives. From the point of view of neuropharmacology, many key neuromodulatory receptors have a laminar specific profile (Eickhoff et al., 2007), which speaks to the importance of understanding the effects of pharmacological agents on population activity within canonical microcircuits. From the perspective of theoretical neurobiology, the role of laminar specific interactions is becoming increasingly important. For example, in predictive coding, the emerging picture suggests that ascending prediction errors may be encoded by superficial pyramidal cell activity and broadcast to higher levels on the cortical hierarchy using gamma frequencies (Bastos et al., 2012). Conversely, descending predictions may be conveyed by lower (e.g. alpha and beta) frequencies from deep pyramidal cells. The (attentional) selection of ascending prediction errors (and descending predictions) rests sensitively on the respective postsynaptic gain of superficial and deep pyramidal cells, which – as we have seen – depends sensitively on interactions with inhibitory interneurons. In short, the synaptic mechanisms that underlie the gain control of neuronal message passing within and between different levels of cortical hierarchies may now be amenable to quantitative study using non-invasive (MEG) procedures.

The ability of the model to explain individual variability in induced responses, and in particular the properties of excitatory and inhibitory sub-populations and intrinsic connections within the model, offers a potentially powerful approach for linking individual variability in behaviour to variability in models of neurophysiology. For example, orientation discrimination performance has previously been linked to variability in GABAergic inhibition (Edden et al., 2009) and it is possible that future work using this type of DCM approach could demonstrate that the CMC model parameters are more sensitive than the data features themselves in terms of explaining individual variability in such tasks.

Our results from Experiment 1, the cohort study, reveal that the most important parameter determining inter-subject variability in peak gamma frequency is the inhibitory self-connection (G7) on superficial pyramidal cells in visual cortex. Furthermore, a contribution analysis shows that increasing self-inhibition suppresses the amplitude of gamma activity, while increasing its peak frequency. This is a key observation that has profound implications for the way we characterise induced responses. In other words, simply summarising induced gamma in terms of their amplitude and frequency overlooks the fact that there is a systematic and nonlinear relationship between these two data features that is mediated by their underlying cause; namely, recurrent inhibition of superficial pyramidal cells. It is only possible to disclose this relationship using forward or generative models of how induced responses are caused.

The fact that self-inhibition of superficial pyramidal cells is the major determinant of observed gamma dynamics is remarkably consistent with theoretical perspectives on neuronal computation in the canonical cortical microcircuit: namely, communication through coherence enables a context-sensitive communication among neuronal populations that may underlie functions such as attention. From the perspective of predictive coding, this context sensitivity (e.g., attention) corresponds to modulating the gain of neurons reporting prediction error, where this gain encodes the precision or uncertainty about the prediction error being reported. Crucially, in neuronal models of predictive coding, prediction error is reported by superficial pyramidal cells. This means that changes in the gain of superficial pyramidal cells encode precision or uncertainty and provides a three-way link between gamma activity, the encoding of precision in predictive coding and attention. This link is entirely consistent with communication through coherence and generalised predictive coding. Furthermore, it highlights the central role of inhibitory control of superficial pyramidal cells in modulating the prediction error (unexplained sensory information) that is passed forward to higher hierarchical levels.

Our finding that tiagabine increases the time constant of inhibitory interneurons shows the remarkable sensitivity of this approach to pharmacological manipulation. The finding is a replication of Thompson and Gahwiler (Thompson and Gähwiler, 1992), who demonstrated that tiagabine increased the decay time-constant of GABA-A receptor mediated synaptic currents in rat hippocampal slices - emphasizing the translational power of this approach. Although the magnitude of the changes we see here are much smaller than the changes observed by Thompson and Gahwiler, this presumably reflects the difference between direct application of tiagabine to the hippocampal slice, compared to relatively modest whole-body dose administrations we performed in humans. In addition, our finding of a mean inhibitory interneuron decay time constant of around 3 ms, in both Experiment 1 and Experiment 2, is consistent with slice recordings from fast-spiking inhibitory interneurons in adult rat neocortex (Galarreta and Hestrin, 2002).

**Fig. 8:**
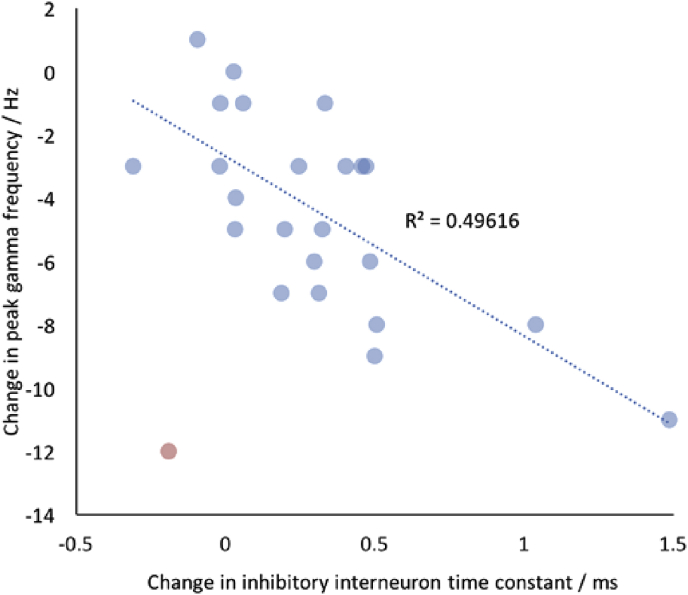
Tiagabine-induced changes in peak gamma frequency are correlated with changes in the modelled inhibitory interneuron time-constant (T3), when pooled across participants and the three post-drug administration sessions. Note one data point (shown in red) is an excluded outlier.

The modelled increase in the inhibitory interneuron time-constant (T3) is also a plausible mechanism for the reduction seen in peak gamma frequency with tiagabine. Peak gamma frequency has been shown in several animal models to be dependent on the time constants of GABAergic inhibition (Xing et al., 2012, Faulkner et al., 1998, Oke et al, 2010, Traub et al., 1996, Whittington et al., 1995). By blocking the reuptake of GABA by GAT-1, tiagabine elevates the synaptic concentrations of GABA (Dalby, 2000, Fink-Jensen et al, 1992) and increases the duration of the GABA_A_ receptor-induced IPSCs (Thompson and Gähwiler, 1992, Roepstorff and Lambert, 1994). These slower IPSCs then result in synchronization of neuronal firing at slower rhythms, which in turn translates to LFP oscillations at lower gamma frequencies. When pooled across participants and post-drug sessions, our results seem to support this model, at least in terms of correlative measures (Fig. 8).

More generally, the demonstrable sensitivity to pharmacological GAT-1 blockade suggests that the use of CMC-based modelling of robust and repeatable oscillatory measures is a powerful new approach for exploring the action of pharmacological agents, including novel compounds, and yields specific information about which synaptic parameters are affected by the drug, together with their pharmacodynamic profile. This could be extremely helpful as part of a drug discovery and evaluation pipeline, helping to reduce the current cost burden of developing new treatments for neurological disease.

In a similar vein, the approach shown here could yield new robust biomarkers for clinical applications within psychiatry, where proposed pathologies are often functional neurochemical deficits in specific networks, which are undetectable with most imaging methodologies (Adams et al., 2013). As such, this method could be crucial, not only for helping to understand the pathology of psychiatric disorders, but in developing pre symptom-onset biomarkers for prevention. Moreover, it holds promise for the stratification of individuals to particular pharmacological treatment groups, since neurotransmitter function can be (DCM) assayed to determine where deficits or targets may exist and appropriate (personalised) medication can then be selected.

## 5 Key limitations

A key limitation of studies employing DCM is that mean-field neural models are, by nature, simplistic compared with the cytoarchitecture of real cortical columns. In the present study, we are guided regarding the trade-off between biological veracity and model estimability by existing work in this field. Indeed the CMC used here is based upon the model and populations in (Muthukumaraswamy et al, 2015, Gilbert et al, 2016, Bhatt et al, 2016, Auksztulewicz and Friston, 2015, Moran et al., 2011b, Pinotsis et al, 2014), differing only in the local coupling among populations. Alterations to the local coupling were implemented in accordance with extant literature (cf. methods). However, it must be noted that this model does represent a likely over-simplification of the true underlying neurophysiology, particularly in the use of a single pooled population of inhibitory interneurons.

Future work may wish to extend the parameter space of these models (at a computational expense and possible lack of stability) to include additional populations in line with specific preclinical findings or hypotheses of regional differences in cortex (e.g. motor cortex (Bhatt et al., 2016)). Similarly, future studies may also wish to extend the model space (and modality of the output) using multi-node models. Indeed this approach has proven useful in modelling network phenomena such as ERPs (see (Garrido et al., 2008)). Extending the model space to include multiple nodes would also preclude the need to explicitly fit alpha as noise, since the number of components in the resulting spectra would be sufficient to fit alpha, beta and gamma simultaneously. Alternatively, the development of more detailed models, which model parameters of cells in more detail than the mesoscopic mean-field approach employed here, would allow greater translation of findings between cellular and imaging neuroscience. Careful consideration of the parameterisation and constraint of such models would be crucial in avoiding over-specification and redundancy. The balance between complexity and estimability can be resolved through validation studies demonstrating that a model has sufficient complexity to accurately recapitulate perturbations. This has been done in preclinical (Moran et al., 2011a, Moran et al, 2014), pharmacological (Muthukumaraswamy et al., 2015) and genetic (identified channelopathies (Gilbert et al., 2016)) studies using 4-population, mean-field CMCs, as used in the present study. Our study adds to this body of work by showing that the GABAergic agent tiagabine, which has a known mechanism of action (from *in-vitro* studies) changes the parameter corresponding to that previously identified *in-vitro* (*T3*; inhibitory interneuron decay time-constant). Thus, we conclude that the CMC employed here is suitably complex for addressing our study aims.

While the equations of motion underlying the CMC employed here are the DCM defaults, we have made changes to the local coupling among populations in line with reports from invasive anatomical studies in the literature. As such, we have not employed a formal model selection routine (e.g. Bayesian model selection) because we wanted the model to be primarily anatomically- and neurophysiologically-informed. Future studied may wish to statistically compare the performance of the CMC given changes in local coupling – however this was outside the remit of the current analysis.

It should be noted that the use of a correlational investigation of the relationship between model parameters and spectral features means that our results are valid only within the context of the model. Furthermore, a formal analysis of the posterior parameter covariances (e.g. principal component analysis) may increase the specificity of parameter-feature correlations, deriving further insight into the generating mechanisms of spectral features.

## Significance statement

Hitherto, most inferences about synaptic function and the effects of pharmacological interventions have been limited to *in-vivo* and in *vitro* recordings. In this study, we show that it is possible to assess synaptic function in terms of rate constants and intrinsic (intra-and inter-laminar) connectivity using non-invasive MEG data from human subjects. This rests upon a careful modelling of how induced or spectral responses are generated. Here we use a canonical microcircuit model to parameterise observed beta and gamma responses in terms of underlying synaptic parameters. This allows one to model, in an empirically constrained fashion, how changing synaptic connections changes observed responses (e.g., gamma). Furthermore, we show how one can track changes in synaptic function over time following pharmacological intervention.
